# Identification of a putative glycosyltransferase responsible for the transfer of pseudaminic acid onto the polar flagellin of *Aeromonas caviae* Sch3N

**DOI:** 10.1002/mbo3.19

**Published:** 2012-06

**Authors:** Jennifer L Parker, Michaela J Day-Williams, Juan M Tomas, Graham P Stafford, Jonathan G Shaw

**Affiliations:** 1 Academic Unit of Immunology and Infectious Diseases, Department of Infection and Immunity, University of Sheffield Medical SchoolSheffield, S10 2RX United Kingdom; 2 Departamento de Microbiología, Facultad de Biología, Universidad de BarcelonaDiagonal 645, 08071 Barcelona, Spain; 3 School of Clinical Dentistry, University of Sheffield, Claremont CrescentSheffield, United Kingdom

**Keywords:** *Aeromonas*, motility, flagella

## Abstract

Motility in *Aeromonas caviae*, in a liquid environment (in broth culture), is mediated by a single polar flagellum encoded by the *fla* genes. The polar flagellum filament of *A. caviae* is composed of two flagellin subunits, FlaA and FlaB, which undergo O-linked glycosylation with six to eight pseudaminic acid glycans linked to serine and threonine residues in their central region. The *flm* genetic locus in *A. caviae* is required for flagellin glycosylation and the addition of pseudaminic acid (Pse) onto the lipopolysaccharide (LPS) O-antigen. However, none of the *flm* genes appear to encode a candidate glycotransferase that might add the Pse moiety to FlaA/B. The motility-associated factors (Maf proteins) are considered as candidate transferase enzymes, largely due to their conserved proximity to flagellar biosynthesis loci in a number of pathogens. Bioinformatic analysis performed in this study indicated that the genome of *A. caviae* encodes a single *maf* gene homologue (*maf1*). A *maf* mutant was generated and phenotypic analysis showed it is both nonmotile and lacks polar flagella. In contrast to *flm* mutants, it had no effect on the LPS O-antigen pattern and has the ability to swarm. Analysis of *flaA* transcription by reverse transcriptase PCR (RT-PCR) showed that its transcription was unaltered in the *maf* mutant while a His-tagged version of the FlaA flagellin protein produced from a plasmid was detected in an unglycosylated intracellular form in the *maf* strain. Complementation of the *maf* strain *in trans* partially restored motility, but increased levels of glycosylated flagellin to above wild-type levels. Overexpression of *maf* inhibited motility, indicating a dominant negative effect, possibly caused by high amounts of glycosylated flagellin inhibiting assembly of the flagellum. These data provide evidence that *maf1*, a pseudaminyl transferase, is responsible for glycosylation of flagellin and suggest that this event occurs prior to secretion through the flagellar Type III secretion system.

## Introduction

Flagella-mediated motility is a common trait among a range of pathogenic bacteria and considered a major virulence factor. Bacterial flagella are complex nanomachines in which a 10- to 15-μm helical filament extends from the cell surface and is anchored to a rotating basal body spanning the bacterial envelope. The helical filament is composed of repeating subunits known as flagellins (Erhardt et al. 2010).

Until relatively recently, it was the accepted dogma that only eukaryotes glycosylated their proteins and that bacteria were unable to do this. However, it has now been demonstrated that several bacterial proteins, especially surface proteins and flagellins, are modified through the linkage to carbohydrate (glycan) groups such as fucose (Fletcher et al. 2011) or various derivatives of sialic acid such as legionaminic or pseud-aminic acid (Logan 2006; Nothaft and Szymanski 2010). Glycosylation of the flagellins, the major subunits of the flagellar filament, has been described for an increasing number of bacteria including *Aeromonas* (Tabei et al. 2009), *Pseudomonas* (Schirm et al. 2004; Verma et al. 2006), *Campylobacter* (Thibault et al. 2001), *Helicobacter* (Josenhans et al. 2002; Schirm et al. 2003), and *Caulobacter* (Leclerc et al. 1998). The role of this glycosylation is not completely understood but is thought to be necessary for flagella filament assembly via its dedicated Type III secretion apparatus with unglycosylated flagellin accumulating in the cytoplasm (Josenhans et al. 2002).

In this study, the opportunistic pathogen *Aeromonas caviae is* employed as a model organism to study the flagella glycosylation system. *Aeromonas caviae* are facultative anaerobic rods that inhabit various aquatic environments. They can cause a number of intestinal and extra-intestinal infections in humans as well as other animals (Janda and Abbott 2010; Parker and Shaw 2011). Mesophilic aeromonads such as *A. caviae* use a distinct lateral flagella system (Laf) for swarming motility over solid surfaces (Kirov et al. 2002), while motility in a liquid environment requires expression of a single polar flagellum made up of two repeating flagellin subunits (FlaA and FlaB) (Rabaan et al. 2001). Our previous studies identified a genetic locus named *flm* in *A. caviae* Sch3N whose encoded products shared homology to proteins involved in polysaccharide biosynthesis or protein glycosylation (Gryllos et al. 2001). Null mutations of five of the genes in the locus (*flmA*, *flmB*, *neuA*, *flmD*, and *neuB*) resulted in a nonmotile aflagellate phenotype (Tabei et al. 2009). The five genes of the locus along with homology data are thus considered the minimum gene set required for pseudaminic acid (Pse5Ac7Ac) biosynthesis (Tabei et al. 2009). Mass spectrometry analysis of purified flagellin indicated that FlaA and FlaB of *Aeromonas* are glycosylated with between six and eight Pse5Ac7Ac moieties linked on to serine and threonine residues in the central immunogenic D2/D3 domains of the flagellin via O-linked glycosylation (Tabei et al. 2009), similar to the flagellin of *Helicobacter pylori* (Schirm et al. 2003). Furthermore, Pse5Ac7Ac was also shown to be present in the lipopolysaccharide (LPS) O-antigen of *A. caviae* Sch3N and two other genes found in the locus, *lsg* and *lst* encoded an LPS-specific transporter and transferase (Tabei et al. 2009). Flagellin glycosylation in *A. caviae* may be considered a prototype system since it encodes only six genes (including *maf*) required for glycosylation of flagellin, while other pathogens such as *Campylobacter jejuni* 81–176 encode many more (up to 30). This is probably due to the fact that *C. jejuni* flagellin is glycosylated with Pse5Ac7Ac and its acetamidino derivative (Pse5Am7Ac), as well as additional glycans (Thibault et al. 2001).

A key step in the glycosylation of flagellin is the transfer of the activated sugar that is linked to cytidine monophosphate (CMP) onto serine and threonine residues within the flagellin central domain. It is likely that such a step would be performed by a flagellin-specific glycosyltransferase enzyme; however, none have been identified to date. Motility-associated factors (Maf proteins) are candidate transferase enzymes for the transfer of glycan molecules on to the flagella, due to their genetic localization and motility phenotypes associated with disruption mutants in other species such as *C. jejuni* (Karlyshev et al. 2002), *A. hydrophila* (Canals et al. 2007), and *H. pylori* (Schirm et al. 2003).

In this study, we examined the function of the single *maf* gene homologue from *A. caviae* Sch3N through insertional mutagenesis and *maf* overexpression followed by extensive phenotypic analysis, and provide evidence that it is a probable flagellin glycosyltransferase that is involved in the transfer of Pse5Ac7Ac onto residues in the central domain of FlaA/B in this organism.

## Materials and Methods

### Bacterial strains, plasmids, and growth conditions

Bacterial strains and plasmids used in this study are listed in Table 1. *Escherichia coli* strains were grown in Luria–Bertani (LB) Miller broth and on LB Miller agar, while *Aeromonas* strains were grown in brain heart infusion broth (BHIB) or on Columbia blood agar (Oxoid). Growth of *E. coli* and *Aeromonas* strains was typically carried out at 37°C. Ampicillin (50 μg/mL), nalidixic acid (50 μg/mL), kanamycin (50 μg/mL), gentamycin (25 μg/mL), streptomycin (50 μg/mL), and chloramphenicol (25 μg/mL) were added to the different media when necessary.

**Table 1:** Strains and plasmids used in this study

Strain or plasmid	Genotype and use or description	Source or reference
*E. coli* strains		
DH5α	F^−^ Phi80*dlacZ* ΔM15 Δ(*lacZYA*-*argF*)U169 *deoR recA1 endA1 hsdR17*(rK-mK+) *phoA supE*44 lambda- *thi*-1; used for general cloning	Invitrogen Life Technologies™ UK
BL21(DE3)	F^−^*ompT hsdSB*(r_B_^−^ m_B_^−^) *gal dcm* (DE3); used to express recombinant proteins in *E*. *coli*	Laboratory stock
S17–1λ*pir*	*hsdR pro recA*, RP4–2 in chromosome, Km::Tn*7* (Tc::Mu) λ*pir*, Tp^r^ Sm^r^	De Lorenzo et al. (1990)
*CC118* λ*pir*	Δ(*ara leu*)*7697 araD139* Δ*lacX74 galE galK phoA20 thi-1 rspE rpoB*(Rf^r^) *argE*(Am) *recA1* λ*pir*^+^	Herrero et al. (1990)
*Aeromonas* strains		
*A. caviae* Sch3N	Sch3, spontaneous Nal^r^	Gryllos et al. (2001)
*A. hydrophila* AH-3	O:34, wild type	Nogueras et al. (2000)
*A. caviae* JPS01	Sch3N; *maf1*::km^r^	This work
*A. caviae* SMT145	Sch3N; *lst::km*^r^	Tabei et al. (2009)
Plasmids		
pGEM	Cloning vector, Amp^r^	Promega, USA
pET28a	Expression vector with hexa-histidine tag, Km^r^	Novagen, Merck International
pUC4KIXX	Source of Tn5-derived *nptII* gene, Km^r^	Pharmacia, GE Healthcare Life Sciences, USA
pKNG101	*ori*R6K *mob*RK2 *strAB sacBR*, 6.8 kb, Sm^r^	Kaniga et al. (1991)
pBBR1MCS	Broad-host-range vector, IncP, -W, -Q, ColE1 and p15A compatible, contains pBluescript IIKS-*lacZα*-polylinker, Cm^r^	Kovach et al. (1994)
pBBR1MCS-5	Broad-host-range vector, IncP, -W, -Q, ColE1 and p15A compatible, contains pBluescript IIKS-*lacZα*-polylinker, Gm^r^	Kovach et al. (1995)
pET28a_*flaA*	pET28 derivative used to express His-tagged fusion of FlaA in *E. coli* BL21(DE3), Km^r^	This work
pSD201	pBBR1MCS-5_*hisflaA*, Gm^r^	This work
pSRK_*hisflaA*	pSRK derivative used to express His-tagged fusion of FlaA in *A. caviae*, Gm^r^	This work
pBBR1MCS_*maf1*	pBBR1MCS with *A. caviae maf1*and 170 bp upstream region, Cm^r^	This work

### General DNA methods

Where required, DNA restriction endonucleases, T4 DNA ligase, and alkaline phosphatase were used as recommended by the suppliers (New England Biolabs Inc, USA).

### Generation of *maf1* disruption mutant

The *maf1* disruption mutant was created by insertion of the Tn*5*-derived kanamycin resistance cartridge (*nptII*) from pUC4-KIXX (Pharmacia, GE Healthcare Life Sciences, USA). For mutation of *maf1*, the 1.4-kb *Sma*I-digested kanamycin resistance cartridge was inserted into a *Sma*I restriction site in the middle of the gene. The *maf1::km* construct was then ligated into the suicide vector pKNG101 (Kaniga et al. 1991) and transferred into *Aeromonas* by conjugation. Conjugal transfer of the recombinant plasmids from *E. coli* S17–1λ*pir* to *A. caviae* Sch3N was performed on Columbia blood agar for 6–8 h at 37°C. Serial dilutions of the mating mixture were then plated on LB agar supplemented with nalidixic acid and kanamycin; the latter antibiotic was added in order to select for recombination. Colonies that were kanamycin resistant (Km^r^) and streptomycin sensitive for pKNG101 derivatives (derivatives not likely to have retained the vector) were selected for analysis by PCR to confirm the mutation prior to phenotypic studies.

### Motility and swarming assays

To assess motility of *Aeromonas* strains, bacterial colonies were transferred with a sterile toothpick into the center of plates containing motility agar (1% tryptone, 0.5% NaCl, 0.3% agar). The plates were incubated face up at 37°C for 14–24 h, and motility was assessed by examining the migration of bacteria through the agar from the center toward the periphery of the plate.

To assess the swarming capabilities of *Aeromonas* strains, bacterial colonies were transferred with a sterile toothpick into the center of plates containing swarming agar (1% tryptone, 0.5% NaCl, 0.5% glucose, 0.002% Tween 80, and 0.8% agar). The plates were incubated face up at 37°C for 24 h, and swarming was assessed by examining the migration of bacteria across the agar from the center toward the periphery of the plate.

### Electron microscopy

Formvar-coated grids were spotted with 1 μl of an *A. caviae* Sch3N overnight BHI culture and allowed to adsorb for 1 min before excess liquid was removed with 3 M blotting paper. Grids were stained with 1% phosphotungstate for 2 min with the excess again removed by blotting. Grids were washed in distilled water and air dried prior to electron microscope analysis. Electron microscopy was carried out using a FEI Tecnai Biotwin 120 Kv transmission electron microscope with a Gatan MS600CW Digital Camera at the University of Sheffield Electron Microscopy Unit in the Department of Biomedical Science.

### LPS extraction and analysis

LPS was extracted from *Aeromonas* strains using an LPS extraction kit (ChemBio Ltd, UK) according to the manufacturer's instructions. Briefly, cells from a 10 mL BHIB culture grown for 16 h were harvested and underwent lysis followed by incubation with chloroform. The supernatant was collected and the LPS purified via precipitation and wash steps. LPS samples were analyzed via Urea-SDS-PAGE (where SDS-PAGE is sodium dodecyl sulphate-polyacrylamide gel electrophoresis) with a 12.5% resolving gel and analyzed by silver staining as previously described (Guard-Petter et al. 1995).

### Cloning and purification of His-tagged flagellin from *A. caviae* and *E. coli* strains

*Aeromonas caviae flaA* was cloned into the protein expression vector pET28a allowing expression of an N-terminal His-tagged fusion of FlaA. For expression in *A. caviae*, the pET28a_*flaA* plasmid was digested with *Xho*I and *Xba*I and the resulting *hisflaA* fragment ligated into the broad-host-range mobilizible vector pBBR1MCS-5 (Gm^r^) cut with the same enzymes generating pSD201. Recombinant *E. coli* BL21(DE3) harboring pET28a_*flaA* and *A. caviae* Sch3N and *maf1-* harboring pSD201 (pBBR1MCS-5_*hisflaA*) were used to inoculate 100 mL of LB or BHIB medium supplemented with appropriate antibiotics and incubated at 37°C with shaking. For BL21(DE3) IPTG was added to a final concentration of 1 mM when cell density reached OD_600_ = 0.5 and growth was continued for an additional 2 h. Growth of *A. caviae* strains was carried out for 16 h. Cells were harvested by centrifugation. The FlaA proteins produced by expression of these constructs possess an N-terminal in-frame 6×His-tag and so facilitate purification by nickel-affinity chromatography. The pellet was resuspended in lysis buffer (20 mM phosphate [pH 7.4], 20 mM imidazol, 0.5 M NaCl, 0.1 mg/mL lysozyme) supplemented with complete protease inhibitor tablets, DNase, and Rnase (Roche) according to the manufacturer's instructions. Cells were disrupted by sonication. The lysate was centrifuged at 27000 *g* for 60 min at 4°C. The supernatant was collected and mixed with an appropriate volume of Ni-NTA superflow resin (Qiagen, UK) that had been equilibrated with *E. coli* lysis buffer and incubated with end over end rotation for 90 min at room temperature. The resin was washed extensively with wash buffer (phosphate buffer [pH 7.4], 0.5 M NaCl, 20 mM imidazole) and eluted with 500 mM imidazole (phosphate buffer [pH 7.4], 0.5 M NaCl, 500 mM imidazol). The presence of the protein of interest was detected by SDS-PAGE and Western blotting.

### SDS-PAGE and immunoblotting

SDS-PAGE and immunoblotting of *Aeromonas* whole-cell preparations was carried out as previously described (Tabei et al. 2009). Briefly, *Aeromonas* strains were grown overnight in BHIB at 37°C. Equivalent numbers of cells were harvested by centrifugation. Cell pellets and protein samples that have undergone nickel-affinity chromatography were boiled in SDS-PAGE loading buffer for 5 min. Protein samples were separated on SDS-polyacrylamide gels (12% acrylamide). For immunoblotting, proteins were transferred onto a Hybond-C (GE Healthcare, USA) nitrocellulose membrane. Following transfer, membranes were blocked with 5% (w/v) powdered skimmed milk. For identification of flagellin, membranes were probed with a polyclonal rabbit antipolar flagellin antibody (1:1500). The unbound antibody was removed by three washes in PBS, and a goat anti-rabbit HRP-conjugated secondary antibody (1:2000) was added. The unbound secondary antibody was washed away with PBS as described above for the primary antibody. For identification of His-tagged flagellin, the membrane was probed with Anti Penta-His (Qiagen, UK product code 34660) (1:1000). The unbound antibody was removed by three washes in PBS, and a goat anti-mouse HRP-conjugated secondary antibody (1:2000) was added and washed and then washed with PBS as described for the primary antibody. The bound conjugate was then detected using the ECL detection system (GE Healthcare, USA). For detection of the ECL signal, the membrane was exposed to X-ray film, adjusting the exposure time to allow for optimization of the signal.

### Transcriptional analysis

To determine that *flaA* transcription occurs in the *maf1* disruption mutant, reverse transcriptase PCR (RT-PCR) was used. Total RNA was isolated using RNeasy Protect Bacteria mini kit (Qiagen, UK) according to the manufacturer's instructions from *A. caviae* strains grown overnight in BHIB. For efficient removal of genomic DNA, an on-column DNaseI digestion was carried out (Qiagen, UK) and following RNA elution from the column a second RNase-free DNaseI (Promega, USA) digestion was used when required.

cDNA generation was carried out using MultiScribe™ Reverse Transcriptase (Invitrogen Life technologies™, UK) with 1 μg of total RNA according to the manufacturer's instructions. Briefly, the RNA was heat denatured for 5 min at 70°C and then incubated at 42°C for 60 min to allow first-strand cDNA synthesis by MultiScribe™ Reverse Transcriptase using the random hexamer primers provided (Invitrogen Life technologies™, UK). Second-strand synthesis and subsequent DNA amplification were carried using the *flaA*- and *16S*-specific primers described in Table 2. The reactions were carried out with an initial enzyme activation step at 95°C for 10 min and then 40 cycles of denaturation at 95°C for 15 sec and primer annealing/extension at 60°C for 60 sec. Amplified DNA was analyzed by agarose gel electrophoresis and resulting bands were subjected to densitometry.

**Table 2:** Primers used in this study

Primer name	Gene/use	Sequence 5’ to 3’ (restriction site)
T7 Promoter	General sequencing of pGEM clones	taatacgactcactata
SP6	General sequencing of pGEM clones	atttaggtgacactatag
Kan right	Mapping the location and orientation of the Kan cassette	tcatttcgaaccccagagtc
Kan left	Mapping the location and orientation of the Kan cassette	tgctcctgccgagaaagtat
JLP_06 F	*A. caviae* Sch3N *maf1* region for disruption	ggatcctgttcatattctattggggca (*Bam*HI)
JLP_07 R	*A. caviae* Sch3N *maf1* region for disruption	ggatccgtatgatgtgtttattaatagg (*Bam*HI)
JLP_82 F	*A. caviae* Sch3N *maf1* + ∼170-bp upstream for complementation	tttaagcttcgtcagattgtccgttcag (*Hind*III)
JLP_31 R	*A. caviae* Sch3N *maf1* for complementation	ggatccttattttttgaatagtacaataacttcattgtc (*Bam*HI)
JLP_84 F	*A. hydrophila* AH-3 *maf1* + ∼170 bp upstream for complementation	tttaagcttgtgtgtttcaacacattgaacttg (*Hind*III)
JLP_85 R	*A. hydrophila* AH-3 *maf1* for complementation	ggatccttaatttattttaaaaacatcaagccctgtg (*Bam*HI)
RT-PCR primers		
16S F	Amplification of 126-bp intragenic region of 16S rRNA	gatccaaccccaggttcccc
16S R	Amplification of 126-bp intragenic region of 16S rRNA	acaccatgggagtgggttgc
*flaA* F	Amplification of 138-bp intragenic region of *flaA*	tttcatcgctcaacgctcagc
*flaA* R	Amplification of 138-bp intragenic region of *flaA*	tcagacggttggaaatctgc

## Results

### *Aeromonas caviae* Sch3N *maf1* is a member of the *maf* family of proteins

**Figure 1:**
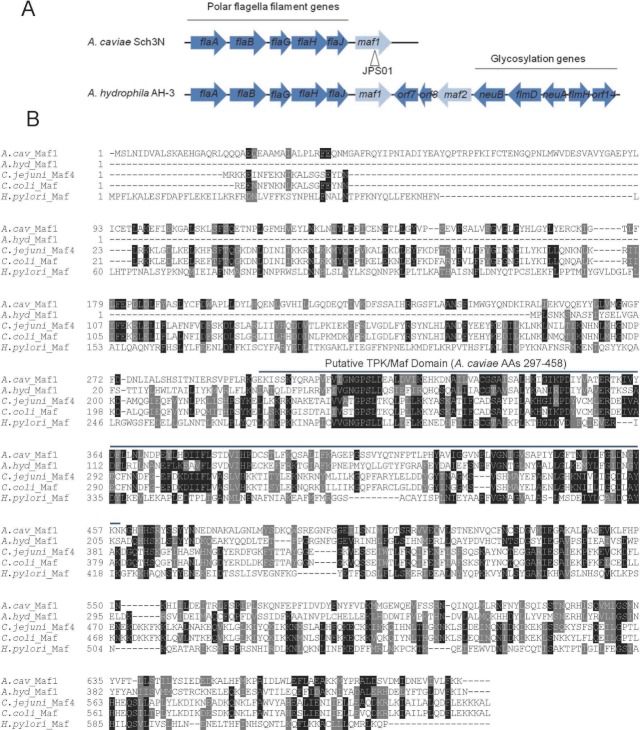
(A) Genetic organization of the polar flagella loci of *Aeromonas caviae* Sch3N and *A. hydrophila* AH-3. Genes known to be involved in flagella filament formation or glycosylation are blue and *maf* genes are gray. The location of kanamycin resistance marker for the generation of the JPS01 insertion mutant (*A. caviae maf1-*) is indicated. (B) Alignment of *A. caviae* Maf1 with homologous Mafs from *A. hydrophila* AH-3 (accession ABA01574), *Campylobacter jejuni* subsp. doyley 269.97 (accession YP_001397580), *C. coli* JV20 (ZP_07400781), and *Helicobacter pylori* 83 (accession AEE70107). The conserved TPK/Maf domain corresponding to *A. caviae* Maf1 amino acids 297–458 is highlighted.

Analysis of the unpublished draft genome sequence of *A. caviae* Sch3N revealed the presence of a single *maf* gene homologue associated with the polar flagellin locus (accession number: JQ254915). Previous work in *A. hydrophila* identified three *maf* homologues, one associated with the lateral flagellin locus (*maf5*), one associated with the polar flagellin locus (*maf1*), and one associated with the flagellin glycosylation locus (*maf2*) (Canals et al. 2007). The organization of the genetic loci containing the polar flagella associated *maf* genes of *A. caviae* Sch3N and *A. hydrophila* AH-3 is shown in Figure 1A.

In a previous study, *maf1* of *A. hydrophila* AH-3 (accession number DQ119104) was annotated as a 1335-bp gene (corresponding to a 444 amino acid protein) (Canals et al. 2007). However, following fresh sequence analysis and complementation studies here, we found that the *A. caviae* Sch3N *maf1* contains an additional N-terminal extension of 700 bp to give a total of 2088 bp, 695 amino acids. Sequence analysis revealed that the *A. caviae Sch3N* single *maf* shared the highest similarity to *maf1* of *A. hydrophila* AH-3 (38% identity) and therefore for clarity we have designated the single *maf* gene of *A. caviae* Sch3N as *maf1*. An alignment of *A. caviae* Sch3N Maf1 with homologous putative Maf proteins of other bacteria is shown in Figure 1B. An NCBI conserved domain database (CDD) search (Marchler-Bauer et al. 2011) (http://www.ncbi.nlm.nih.gov/Structure/cdd/wrpsb.cgi) using the Maf1 protein sequence revealed the presence of a single putative domain that shows homology to members of the thiamine pyrophosphokinase (TPK) superfamily, the location of which is shown in Figure 1B. Thiamine pyrophosphokinases catalyze the transfer a pyrophosphate group from a nucleoside triphosphate, such as ATP, to the hydroxyl group of thiamine producing thiamine pyrophosphate. The role of this domain in Maf function is unknown but its high conservation with Mafs from *A. hydrophila*, *C. jejuni*, *C. coli*, and *H. pylori* (Fig. 1B) indicates its importance. While there are no reports of this domain being detected in other glycosyltransferase proteins by sequence homology searching, there is a structural homology between the lipooligosaccharide-specific sialyltransferase CstII from *C. jejuni* and mouse thiamine pyrophosphokinase, indicating that this might have a role in the catalytic mechanism of protein glycosylation in bacteria (Chiu et al. 2004).

A key step in flagellin glycosylation is the transfer of activated glycans (CMP-Pse5Ac7Ac for *A. caviae*) to the hydroxyl group of serine and threonine residues in the central D2/D3 domain of flagellin via O-linked glycosylation. Such a step would be performed by a flagellin glycosyltransferase that we hypothesized would be Maf1 in *A. caviae* Sch3N.

### *Aeromonas caviae* Sch3N *maf1* is required for polar flagella-mediated motility

To determine the roles of the identified *maf1* gene, a disruption mutant was constructed. A kanamycin resistance cassette was inserted in the same transcriptional orientation with respect to the target gene (Fig. 1A); the presence of an outward-reading promoter on the cassette ensures expression of downstream genes, thereby reducing any polar effects. However, the genes located downstream of *maf1* (including the putative periplasmic binding protein AHA1704) are transcribed in the opposite orientation, meaning any polar effects are unlikely (Fig. 1A). The construction of the mutant was verified by PCR using primers specific for the *maf1* gene and the kanamycin resistance cassette (Table 2) in combination, so as to confirm both the location and orientation of the insertion (data not shown).

**Figure 2:**
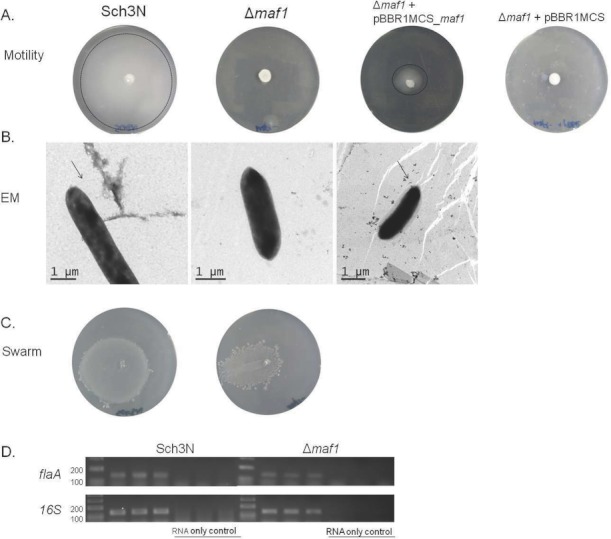
(A) Analysis of motility of the *Aeromonas caviae maf1* mutant JPS01 and derivative strains. Motility as assessed by swimming in 0.25% semisolid motility agar for *A. caviae* Sch3N (WT), JPS01 (*maf1* mutant), and JPS01 containing pBBR1MCS_*maf1* and pBBR1MCS. (B) Transmission electron microscopy of the *A. caviae* strains Sch3N (wild type), *maf1*-, and *maf1*- + pBBR1MCS_*maf1* grown at 37°C in brain heart infusion broth (BHIB). Bacteria were spotted onto Formvar-coated copper grids and negatively stained using 1% phosphotungstate. (C) Analysis of swarming motility of the *maf1* mutant as assessed by movement across the surface of swarming agar. (D) Reverse transcriptase PCR (RT-PCR) analysis of *flaA* gene expression of *A. caviae* Sch3N (WT) and *maf1*-. Primers internal to 16S rRNA gene of *A. caviae* were used as a control. Experiments were performed in triplicate. Primer pairs are listed in Table 2.

In order to test the effect of disruption of the *maf1* gene on motility of *A. caviae*, its ability to migrate through semisolid agar on motility plates (0.25% w/v agar) was assessed. The *maf1-* mutant was unable to swim in this semisolid motility agar, unlike the wild-type strain Sch3N that migrated to the edge of the plate in the same time period (Fig. 2A). Electron microscopy analysis of negatively stained *A. caviae* wild-type and *maf1* strains with 1% phosphotungstate revealed that the *maf1-* completely lacked polar flagella, whereas the single polar flagellum was clearly visible in the wild-type strain (Fig. 2B). Motility of the *maf1*- strain over solid surfaces, also known as swarming motility, via the distinct lateral flagella system still proceeded at approximately the same rate as the wild-type strain but displayed a more irregular swarm colony than the wild-type strain (Fig. 2C). This indicates that while *maf1* is not essential for swarming motility, as it is for polar flagella-mediated motility, it may still play an as yet unknown role.

For complementation of *maf1*-, *maf1* plus approximately 170 bp upstream (predicted to encompass its native promoter) was cloned into the multicopy broad-host-range mobilizable vector pBBR1MCS (Cm^r^). This construct was introduced into the *maf1-* strain by conjugation. Re-introduction of *maf1* in trans partially restored motility of the mutant to approximately 50% of wild-type levels (Fig. 2A). While this is clearly not complete complementation, transmission electron microscopy analysis revealed that addition of pBBR1MCS_*maf1* restored the formation of the single polar flagellum (Fig. 2B). The partial complementation of the motility phenotype is likely due to the multicopy nature of pBBR1MCS providing increased copies of *maf1*, resulting in an overexpression phenotype rather than complementation. A low copy number plasmid may provide a better complementation, however such a plasmid is not available for use in *A. caviae*.

In order to confirm that *flaA* is successfully transcribed in the *maf1* mutant and that the nonmotile phenotype is not due to any regulatory feedback mechanisms on the *flaAB* operon, RT-PCR analysis of *flaA* gene expression of *A. caviae* Sch3N (WT) and the *maf1*- strain was performed. The wild-type and *maf1*- mutant strain was grown overnight in BHIB at 37°C and RNA was extracted. The presence of the *flaA* transcript was confirmed in the *maf1*- strain (Fig. 2D). Gel bands were analyzed by densitometry and results showed that the *flaA* transcript levels found in the *maf1* mutant were at levels comparable to that of the wild type, indicating that *flaA* transcription is unaffected in the *maf1*- strain.

### *Aeromonas caviae* Sch3N *maf1* is a putative flagellin pseudaminyl transferase

**Figure 3:**
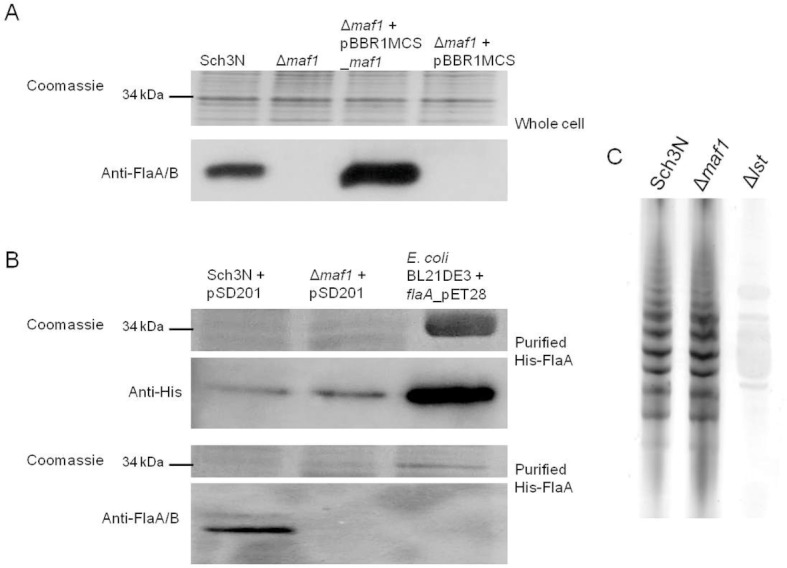
(A) Western blot analysis of whole-cell protein preparations from the *Aeromonas caviae* wild-type and *maf1* mutant strain using polyclonal rabbit antipolar flagellin antibodies (1:1000). Lane 1, Sch3N (wild type); lane 2, *maf1*-; lane 3, *maf1*- + pBBR1MCS_*maf1*; lane 4, *maf1-* + pBBR1MCS. Whole-cell proteins were obtained from bacteria grown at 37°C in brain heart infusion broth (BHIB). (B) Western blot analysis of His-tagged flagellin protein preparations from *A. caviae* wild-type and *maf1*-harboring pSD201 (pBBR1MCS-5_*hisflaA*), and *E. coli* BL21(DE3) harboring pET28a_*flaA* probed with anti-Penta-His antibodies (panel 1) and anti-FlaA/B antibodies (panel 2). Lane 1, Sch3N + pSD201, lane 2, *maf1*- + pSD201; lane 3, BL21(DE3) + pET28a_*flaA*. (C) Analysis of lipopolysaccharide (LPS) isolated from *A. caviae* Sch3N (WT), JPS01 (*maf1-*), and SMT145 (*lst*-). LPS was extracted from bacteria grown at 37°C in BHIB, analyzed by SDS-PAGE (12%), and silver stained.

To investigate whether *maf1* has an effect on the glycosylation status of FlaA/B, Western blot analysis was carried out on whole-cell preparations of *A. caviae* WT, *maf1*-, and the *maf1* complementation strain using an anti-FlaA/B antibody that has a high affinity for glycosylated but not unglycosylated flagellin (Rabaan et al. 2001; Tabei et al. 2009) (Fig. 3A). We favor the use of whole-cell blots for analysis of flagellin expression in *A. caviae* since we have found traditional shearing and ultracentrifugation protocols for the concentration of flagella inconsistent in *A. caviae*, probably due to circumstantial evidence that the filaments are more fragile than in enteric strains. Results revealed that glycosylated *A. caviae* polar flagellin could not be detected in the *maf1*- strain (Fig. 3A, lane 2). In contrast, the *maf1* complemented strain displayed glycosylated flagellin levels higher than that of the wild type (Fig. 3A, lane 3), this was surprising given that it did not fully restore motility (Fig. 2A).

The most likely explanation for this result is that the glycosylation status of FlaA/B is altered to such an extent in the *maf1*- mutant that the anti-FlaA/B antibody does not recognize it even though it is still present. However, it is also possible that it is undetected due to instability of the flagellin protein in the absence of *maf1*. However, since we do not have an antibody that detects unglycosylated *A. caviae* flagellin, we cloned *flaA* into the broad-host-range vector pBBR1MCS-5 with an N-terminal His-tag attached for expression in *A. caviae*, and into pET28a for expression in *E. coli* BL21(DE3). BL21(DE3) acts as a control for the production of unglycosylated *A. caviae* flagellin as it lacks any Pse5Ac7Ac biosynthesis or flagellin glycosylation systems. Production of His-tagged flagellin would allow detection of nonglycosylated FlaA in *A. caviae. Aeromonas caviae* Sch3N and *maf1*- both harboring pSD201 (pBBR1MCS-5_*hisflaA*), and *E. coli* BL21(DE3) harboring pET28a_*flaA* were grown at 37°C and protein expression was induced in *E. coli* BL21(DE3) harboring pET28a_*flaA* with the addition of IPTG (1 mM final concentration) while expression in *A. caviae* was allowed to occur at basal levels in the noninducible pBBR1MCS-5 plasmid. *Aeromonas caviae* and *E. coli* cells were harvested by centrifugation, lysed, and the soluble fraction submitted to nickel-affinity chromatography. Resulting preparations of His-FlaA from wild type, *maf1*-, and *E. coli* BL21(DE3) underwent analysis by SDS-PAGE and Western blotting using an anti-His antibody (Fig. 3B, panel 1) and the anti-FlaA/B antibody (Fig. 3B, panel 2). Bands corresponding to His-FlaA were visible in the wild-type, *maf1*-, and BL21(DE3) strains when probed with anti-His (Fig. 3B, panel 1). In contrast, glycosylated flagellin was undetected in the same samples when probed with anti-FlaA/B (Fig. 3B, panel 2). This confirms that our anti-FlaA/B antibody recognizes and binds the glycosylated form of FlaA/B purified from *A. caviae* Sch3N with much higher affinity than the nonglycosylated form purified from *E. coli* and that flagellin is indeed present but found unglycosylated in the absence of a functional *maf1* gene. There was a slight increase in the amount of nonglycosylated flagellin recovered from the *maf1-* strain, indicating that the flagellin is not exported or assembled. Higher levels of His-FlaA were also seen in *E. coli* BL21(DE3) where expression of His-FlaA was induced to very high levels with the addition of IPTG (Fig. 3, panel 1). With FlaA and FlaB sharing 92% identity at the amino acid level, and both flagellin subunits previously being shown to be modified with Pse5Ac7Ac (Tabei et al. 2009), we would also predict that FlaB is found unglycosylated in the absence of *maf1*, particularly as no glycosylated polar flagellin could be detected in whole-cell blots of *maf1*- (Fig. 3A).

Along with glycosylated flagellin, Pse5Ac7Ac is present on the LPS O-antigen of *A. caviae* Sch3N, with disruption of any of the genes in the Pse5Ac7Ac biosynthetic pathway (*flmA*, *flmB*, *neuA*, *flmD*, and *neuB*) leading to the loss of Pse5Ac7Ac from the LPS (Tabei et al. 2009). In order to confirm that Maf1 is not required for Pse5Ac7Ac biosynthesis, we investigated whether or not *maf1* disruption has an effect on LPS O-antigen synthesis. The wild-type, *maf1*- mutant strain, and the *lst*- mutant strain (LPS O-antigen) were grown overnight in BHIB at 37°C, and LPS was extracted and analyzed by PAGE. Both the wild-type and *maf1*- strains produced the same LPS pattern indicating that disruption of *maf1* has no effect on the assembly of the LPS O-antigen (Fig. 3C), and is therefore not involved in Pse5Ac7Ac and LPS biosynthesis. Taken together, these data provide evidence that Maf1 is involved in flagellin glycosylation but not required for Pse5Ac7Ac biosynthesis and therefore may be a pseudaminyl transferase specifically responsible for the transfer of Pse5Ac7Ac onto FlaA/B of *A. caviae*.

### Overexpression of Maf1 has a detrimental effect on motility

**Figure 4:**
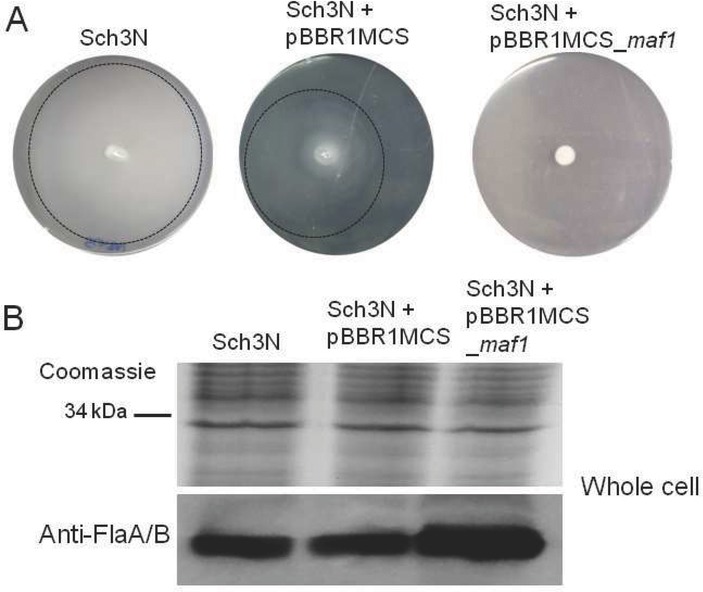
(A) Analysis of motility of *Aeromonas caviae* Sch3N, Sch3N + pBBR1MCS, Sch3N + pBBR1MCS_*maf1*, and *maf1*- + pBBR1MCS_AH3 *maf1*. Motility as assessed by swimming in 0.25% semisolid motility agar. (B) Western blot analysis of whole-cell protein preparations from the *A. caviae* wild-type and *maf1* overexpression strain using polyclonal anti-polar flagellin antibodies (1:1000). Lane 1, Sch3N (wild type); lane 2, Sch3N + pBBR1MCS; lane 3, Sch3N + pBBR1MCS_*maf1*. Whole-cell proteins were obtained from bacteria grown at 37°C in brain heart infusion broth (BHIB).

To further investigate as to why the increased levels of glycosylation detected in the *maf1* complemented strain (Fig. 3A, lane 3) only partially restored motility (Fig. 2A), we tested the hypothesis that this was causing a dominant negative effect rather than a lack of complementation of the *maf1-* strain. It is possible that the phenotype exhibited by the *maf1* complemented strain could be due to an oversupply of glycosylated FlaA/B to the flagellar Type III secretion system, alternatively the increased levels of Maf1 could cause nonspecific glycoylation of flagellin, affecting its secretion. The plasmid pBBR1MCS_*maf1* was transferred into Sch3N (WT) for overexpression of *maf1*, and motility was assessed. Motility assays showed that Sch3N + pBBR1MCS_*maf1* was nonmotile, whereas the transfer of the empty pBBR1MCS plasmid had very little effect on motility (Fig. 4A). This result indicates that a particular level of Maf1 protein is required for optimal motility and increasing it considerably more than the wild-type levels, as we have done here by attempting complementation using the multicopy pBBR1MCS vector, has a detrimental effect on flagella motility. Western blot analysis of Sch3N + pBBR1MCS_*maf1* revealed that higher levels of intracellular glycosylated flagellin were present when *maf1* was overexpressed compared to the wild-type Sch3N (Fig. 4B), although this strain was found to be nonmotile.

## Discussion

In this study, we identified the presence of a single putative *maf* gene in *A. caviae*, which was annotated *maf1*. Maf proteins show no homology to flagella structural proteins, and are associated with flagellin glycosylation islands. Disruption of *maf1* resulted in nonmotile aflagellate cells that retained swarming activity via the distinct lateral flagella system. However, it was observed that the swarm colonies had a more irregular pattern at the edges of the swarm front than those of the wild-type colonies. Mutation of *maf1* had no effect on LPS O-antigen synthesis, indicating that *maf1* has a specific role in polar flagella-mediated motility. These data are in contrast to the more complex situation in *A. hydrophila* where there are two putative *maf* genes in the polar flagella locus (*maf1* and *maf2*) and one in the lateral flagella locus (*maf5*). Previous investigations into the role of *maf2* of *A. hydrophila* confirmed that it is required for both polar and lateral flagella-mediated motility (Canals et al. 2007). Here, we confirm using western blots that *A. caviae* polar flagellin is glycosylated in the D2/D3 central domain with between six and eight Pse5Ac7Ac residues (Tabei et al. 2009) by the action of *maf1* only. There was also an increase in the amount of nonglycosylated flagellin in the *maf1-* strain, since in its unglycosylated form it has been previously shown that flagellin is not exported or assembled (Goon et al. 2003). Our data also highlight that that there was little effect on swarming motility via the lateral flagella system in the *maf1*- mutant strain, even though previous work confirmed that the *A. caviae* lateral flagellin (LafA1/LafA2) displays aberrant migration when analyzed by SDS-PAGE after periodate and hydrazine treatment, and is likely to be posttranslationally modified through O-linked glycosylation (Gavin et al. 2002). This either indicates that *maf1* is not required for lateral flagellin glycosylation, or that pseudaminlyation of LafA1/LafA2 via Maf1 is not essential for swarming motility.

**Figure 5:**
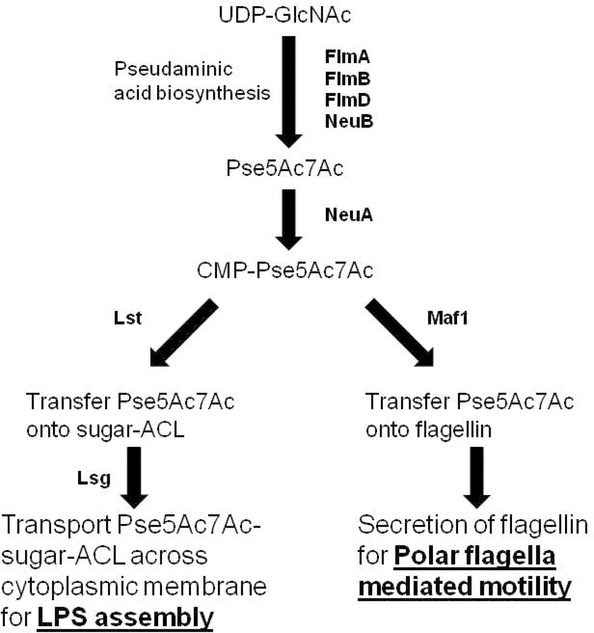
Hypothetical pathway for flagellin glycosylation and lipopolysaccharide (LPS) modification in *Aeromonas caviae* Sch3N. The biosynthetic pathway to Pse5Ac7Ac is based on the predicted functions of the *A. caviae* proteins compared with those elucidated for *Campylobacter jejuni* and *Helicobacter pylori* proteins (McNally et al. 2006; Schoenhofen et al. 2006). Following biosynthesis of Pse5Ac7Ac by the FlmABD and NeuB, Pse5Ac7Ac is activated by covalent linkage to CMP with NeuA. CMP-Pse5Ac7Ac is then either transferred onto the flagellin by Maf1, which we predict to be a polar flagellin specific glycosyltransferase, or transferred onto a sugar-antigen carrier lipid (ACL) by Lst to create an LPS O-antigen unit, and this O-antigen unit is subsequently transported across the cytoplasmic membrane by Lsg.

Disruption of *maf1* having no effect on LPS O-antigen biosynthesis confirmed that Maf1 does not play a role in Pse5Ac7Ac biosynthesis or activation, just as disruption of *lsg* or *lst* revealed that they are specifically involved in LPS bio-synthesis and do not affect flagella formation or Pse5Ac7Ac biosynthesis (Tabei et al. 2009). Taken together, these results suggest that *maf1* is required for flagellin glycosylation following Pse5Ac7Ac biosynthesis, after the activation of Pse5Ac7Ac to CMP-Pse5Ac7Ac. Our model for this pathway is outlined in Figure 5. In light of our new data, we propose for the first time that Maf proteins are likely to be a new family of glycosyltransferases responsible for transfer of activated glycans onto flagellin. This is further supported by other studies in bacteria encoding multiple putative *maf* genes. *Campylobacter jejuni* encodes seven putative *maf* genes (Karlyshev et al. 2002). Mutations of these *mafs* have been shown to alter the glycosylation pattern and that expression of several of the Maf proteins in *Campylobacter* is switched on/off by phase variation events, resulting in the flagellins being decorated with different sugars (Karlyshev et al. 2002). Taken together with the evidence described in this study, we would also hypothesize that the number of *maf* genes directly corresponds to the diversity of sugars employed to modify flagellin. Interestingly, our attempts to complement *A. caviae maf1*- nonmotile phenotype with *A. hydrophila* AH-3 *maf1* failed to restore motility (Data not shown), indicating that *A. hydrophila* flagellin may be modified with a glycan other than pseudaminic acid.

As far as we know, this is the first example of a bacterium that encodes a single *maf* gene homologue that is specifically involved in polar flagellin-mediated motility via glycosylation, with no affect on LPS O-antigen biosynthesis and little affect on lateral flagella mediated motility. This could reflect the finding that *A. caviae* flagellin is homogenously glycosylated with Pse5Ac7Ac residues (Tabei et al. 2009) and therefore only a single glycosyltransferase/Maf is required for the key step of transfer of the glycan to specific serine and threonine residues after its synthesis. The fact that *A. caviae* has a single *maf* gene and the flagellin is homogenously glycosylated makes it an ideal model organism to study the mechanism of flagellin glycoylation in pathogenic bacteria.

To our knowledge, this is the first report of a dominant negative effect of Maf when provided on a multicopy plasmid and showed that overexpression of *maf1* in the wild-type Sch3N abolished motility yet contained higher levels of intracellular glycosylated FlaA/B than the wild type. It is possible that this dominant negative effect is possibly caused by these high amounts of glycosylated flagellin resulting from the *maf1* overexpression, perhaps accumulating at the basal body earlier than required or at too high level, subsequently inhibiting assembly of the flagellum. Previous studies have indicated that overproduction of basal-body proteins FlgB, FlgC, FlaF, and FlaG in *Salmonella* can inhibit motility indicating that timing and levels of T3SS components are crucial (Hirano et al. 2003). Further bioinformatic analysis using the subcellular localization prediction software SOSUI-GramN (Imai et al. 2008) (http://bp.nuap.nagoya-u.ac.jp/sosui/) predicts that *A. caviae* Maf1 is located in the cytoplasm, indicating that the process of transferring activated Pse5Ac7Ac onto the flagellin occurs in the cytoplasm prior to flagellin export.

Although we have not provided direct evidence of Maf1 showing glycosyltransferase activity, taken together, our data provide strong evidence that Maf proteins may form a novel family of flagellin glycosytransferases that warrant further study, for example, what is their mechanism of action? What is the role of the TPK domain? And what is the exact sequence of events with regard to flagellin glycosylation? These questions are currently under investigation in our laboratories.
